# Imaging appearance of isolated diffuse neurofibroma of nipple areolar area: a case report

**DOI:** 10.11604/pamj.2021.39.178.30438

**Published:** 2021-07-07

**Authors:** Siham Nasri, Youssef Benmoussa, Widad Abbou, Houda Mirali, Narjiss Aichouni, Imane Skiker

**Affiliations:** 1 Mohammed VI University Hospital Center, Faculty of Medicine and Pharmacy, Mohammed I University, Oujda, Morocco

**Keywords:** Nipple areolar area, neurofibroma, mammography, case report

## Abstract

Sporadic neurofibromas of the nipple-areolar complexes are exceptional even in patients with neurofibromatosis. Diffuse neurofibroma is an uncommon subtype of neurofibroma that has received little attention in the imaging literature. As are most superficial lesions, it is often evaluated clinically and if biopsy is needed, it is usually performed without imaging. However the imaging data is quite characteristic with the aim of evaluating the extension in depth and detecting an underlying cancer. We report a case of women without a history of neurofibromatosis presenting a skin thickening disfiguring her left breast, related to diffuse neurofibroma of the nipple-areolar complexes confirmed histologically. We study echo-mammography and breast magnetic resonance imaging (MRI) findings in order to highlight its radiographics features.

## Introduction

Neurofibromas are defined as histologically benign (WHO grade I) tumors [1]. It is a rare tumor, usually in the mesodermal or neuroectodermal tissues of head and neck, although any organ or system can be primarily or secondarily involved. They originate in the external nerve sheath and are characterized by the proliferation of Schwann cells, perineural cells and endoneural fibroblasts [2]. The most common and prominent location of neurofibromas is the skin (epidermis and dermis). The anatomical sites of predilection for neurofibromas include the trunk, extremities, head, and neck. They can be sporadic or neurofibromatosis-associated [3]. We present a sporadic and “special” anatomical site of diffuse neurofibroma that is nipple areolar area.

## Patient and observation

**Patient information:** a 29 years-old, married woman, presented with massive involvement of the aspect of the skin that disfiguring her right breast. After her last childbirth and in the past several years she had noted further enlargement of the skin of her left nipple-areolar region, the lesion was painful and it was so disturbing to her that she was unable to carry on normally. Her medical history was unremarkable, and there were no family history of neurofibromatosis.

**Figure 1:**
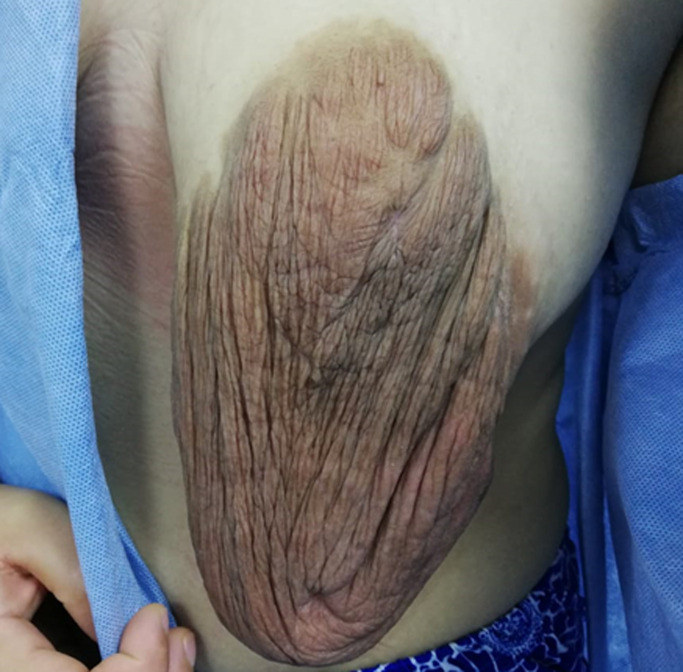
overgrowth of the cutaneous tissues of left nipple-areolar region

**Clinical findings:** a systematic multidisciplinary clinical investigation and familial enquiry were performed. Examination revealed a large and extended outward 8cm lesion of the skin of her left nipple-areolar complex which markedly deformed it (Figure 1), without inflammatory sign or a palpable nodule in the gland. The hue abnormalities were dark brown. The right nipple-areolar area was normal and she had no *café-au-lait* spots in her skin.

**Figure 2:**
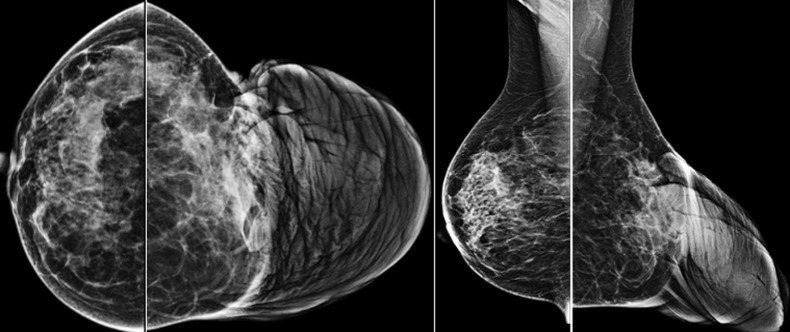
mammography: extensive cutaneous and subcutaneous soft tissue mass that disfiguring the left breast

**Figure 3:**
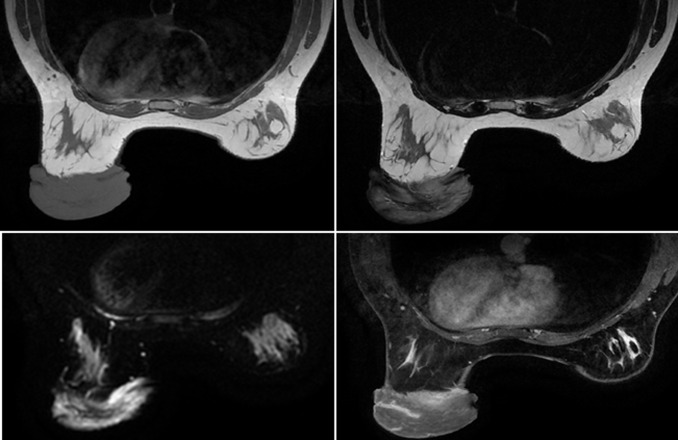
plaque-like elevation of the skin with thickening of the entire subcutis but without nodular masses. Iso-intense to skeletal muscle on T1, hyperintense on T2 with contrast enhancement after injection of gado

**Diagnostic assessment:** mammography and ultrasound showed a hypertrophy of the cutaneous tissue of the left areolar plate of homogeneous hypoechoic echostructure. Without suspicious cystic or tissue intraglandular lesion (Figure 2). Free lymph node areas. In the case of a pass next to an underlying cancer, a breast MRI was performed with sequences weighted in T1, T2, T2 FATSAT, STIR, diffusion and T1 FATSAT after injection of Gadolinium in dynamic mode with sequences of substraction. It revealed significant skin thickening of the areolo-nipple plaque, isointense in T1, heterogeneous T2 hypersignal, diffusion hypersignal enclosing enhanced spans after contrast (Figure 3). Skin biopsy showed the appearance of a neurofibroma confirmed by an immunohistochemical study. A brain and orbital MRI was done as part of the search for associated neurofibromatosis coming back negative.

**Therapeutic intervention and follow-up:** the patient underwent lumpectomy with breast reconstruction. The postoperative consequences were simple. The anapath of the nipple-areolar plaque resection piece confirmed the diagnosis of a diffuse neurofibroma. Resection limits was healthy. There was no recurrence with two years follow-up.

**Patient perspective:** the patient was satisfied of the treatment and the follow-up she received.

**Informed consent:** the patient gave informed consent.

## Discussion

Three types of neurofibroma are classically described: localized, diffuse, and plexiform. Localized and plexiform neurofibromas are well known subtypes: the first one is the most common representing 90% of these lesions. Clinically they are skin-colored dome-shaped or pedunculated papules that display the pathognomonic “button hole” sign. It is the subtype most familiar to radiologists because its imaging appearance has been well documented [2-4]. Plexiform neurofibromas diffusely involve single or multiple nerve branches [2]. Clinically, these tumors often present as a superficial cutaneous or subcutaneous lesion, but may occur in all parts of the body, imparting a sensation of “a bag of worms” on palpation. These tumors are usually diagnosed in childhood and are associated with neurofibromatosis 1 (NF1) in about 30% [5].

Diffuse neurofibroma is an uncommon subtype of neurofibroma that has received little attention in the imaging literature. They are usually single lesions, although It is suggested that 10% of patients with diffuse neurofibromas have NF1 [2]. Unlike other types of neurofibroma, which have a mass like pattern of growth, diffuse neurofibroma is a variably size and ill-defined lesion which diffusely infiltrate the dermis and subcutaneous tissues, which appear like a thickening of those [4]. It has been reported to occur in the head and neck regions of children and young adults. Recent series reported the trunk and extremities as the commonest locations [2]. The localization at the breast is exceptional even for patients with NF1. Bongiorno *et al*. reported only nine out of 258 patients, or 3.45% of the total number of the patients evaluated with a diagnosis of NF1 in their dermatology department harboured neurofibromas of the breast [6].

The purpose of imaging is to determine the extent of large lesions and those suspected of having deeper extension. Cross-sectional imaging, including MRI, computed tomography (CT), and sonography, is well suited to defining the extent of disease. Imaging features most suggestive of diffuse neurofibroma include a plaque like or infiltrative pattern of growth involving the skin and subcutaneous tissues, prominent internal vascularity, and marked contrast enhancement [7]. Sonography has been increasingly used in the evaluation of soft-tissue masses, including diffuse neurofibroma. Wide availability, lack of ionizing radiation, cost-effectiveness, and speed of examination are advantages of sonography. Sonography, however, can be limited in assessment of the extent of large lesions and in discerning lesion margins. Diffuse neurofibroma is hyperechoic permeated by multiple interconnecting irregular hypoechoic tubular or nodular structures whether it was located in the subcutaneous fat zone or the subcutaneous plaquelike portion.

Magnetic resonance imaging reveals a characteristic extensive infiltration of skin and subcutaneous fat that envelops vessels and tendons. Neurofibroma tissue is isointense or mildly hyperintense in relation to muscle on T1-weighted images and mildly or markedly hyperintense to muscle on T2 weighted images. Prominent internal vascularity and homogeneous enhancement following intravenous gadolinium administration are mostly found [4-7]. On histopathological examination, diffuse neurofibromas are ill-defined tumors that diffusely infiltrate the dermis and subcutaneous tissue, that spreads along connective tissue septa and surrounds rather than destroys adjacent normal structures. They are composed of elongated spindle-shaped cells with round to fusiform nuclei and eosinophilic cytoplasm within a loose matrix of fibrillary collagen. Meissner bodies, a characteristic feature of diffuse neurofibromas, are not always present. Fat and ectatic blood vessels may be dominant features. Expression of S-100 protein is characteristic and a sensitive, but non-specific, marker [2].

Complications of diffuse neurofibromas are limited limb movements, neurological deficits, bleeding due to trauma, malignant transformation and disfigurement. It often causes cosmetic and functional disturbances, resulting in impaired quality of life [1]. The association of neurofibromas of the breast and a breast cancer especially in patient with NF1 is possible. The finding that both the NF1 gene and a breast cancer predisposition gene (BRCA1) are located in close proximity on chromosome 17q makes the association of these two conditions intriguing in addition presence of multiple neurofibromas of the breast, which can develop both on the surface of the skin and subcutaneously, may obscure a breast mass at palpation, leading thus to more rigorous clinic and mammographic screening of the breast during adulthood to determine the presence or absence of malignancies [5].

## Conclusion

Neurofibromas are rare, benign, peripheral nerve sheath tumours. They typically develop in the context of neurofibromatosis on the trunk or limbs. Three types of neurofibroma are classically described: localized, diffuse, and plexiform. Sporadic diffuse neurofibroma of the breast is exceptional with a characteristic imaging appearance. Imaging features most suggestive include infiltrative pattern of growth involving the skin and subcutaneous tissues and marked contrast enhancement.
